# Sleep disturbances and racial–ethnic disparities in 10-year dementia risk among a national sample of older adults in the USA

**DOI:** 10.1192/bjo.2024.814

**Published:** 2024-12-04

**Authors:** Roger Wong, Jason Rafael Grullon

**Affiliations:** Department of Public Health and Preventive Medicine, Norton College of Medicine, SUNY Upstate Medical University, New York, USA; and Department of Geriatrics, SUNY Upstate Medical University, New York; USA; Norton College of Medicine, SUNY Upstate Medical University, New York, USA

**Keywords:** Sleep–wake disorders, dementias/neurodegenerative diseases, medication, health disparities, race

## Abstract

**Background:**

Race/ethnicity and sleep disturbances are associated with dementia risk.

**Aims:**

To explore racial–ethnic disparities in sleep disturbances, and whether race/ethnicity moderates the relationship between sleep disturbances and dementia risk among older adults.

**Method:**

We analysed ten annual waves (2011–2020) of prospective cohort data from the National Health and Aging Trends Study, a nationally representative USA sample of 6284 non-Hispanic White (*n* = 4394), non-Hispanic Black (*n* = 1311), Hispanic (*n* = 342) and non-Hispanic Asian (*n* = 108) community-dwelling older adults. Sleep disturbances were converted into three longitudinal measures: (a) sleep-initiation difficulty (trouble falling asleep within 30 min), (b) sleep-maintenance difficulty (trouble falling asleep after waking up early) and (c) sleep medication usage (taking medication to sleep). Cox proportional hazards models analysed time to dementia, after applying sampling weights and adjusting for sociodemographic characteristics and health.

**Results:**

Black, Hispanic and Asian respondents exhibited higher frequencies of sleep-initiation and sleep-maintenance difficulties, but had less sleep medication usage, compared with White older adults. Among Hispanic respondents, sleep-initiation difficulty was associated with significantly decreased dementia risk (adjusted hazard ratio (aHR) = 0.34, 95% CI 0.15–0.76), but sleep-maintenance difficulty was associated with increased dementia risk (aHR = 2.68, 95% CI 1.17–6.13), compared with White respondents. Asian respondents using sleep medications had a significantly higher dementia risk (aHR = 3.85, 95% CI 1.64–9.04). There were no significant interactions for Black respondents.

**Conclusions:**

Sleep disturbances are more frequent among older Black, Hispanic and Asian adults, and should be considered when addressing dementia disparities. Research is needed to explore how certain sleep disturbances may elevate dementia risk across different racial and ethnic subgroups.

Recent studies have identified sleep disturbances as a potential contributor to dementia risk.^1,2^ Although the definitive mechanism for dementia remains elusive, evidence suggests a possible sleep disturbance component in dementia aetiology.^3^ For example, when rodents are sleep-deprived, there is a significant increase in dementia-related biomarkers.^4^ In healthy adults, evidence has shown that one night of sleep deprivation can significantly increase amyloid-β burden in the hippocampus.^5^ Moreover, when specific human brain waves are experimentally suppressed disrupting deep non-rapid eye movement (REM) sleep, amyloid-β levels in cerebrospinal fluid can become significantly elevated.^6^ Disrupted sleep can even serve as a driver for brain-wide disease progression by influencing the pathological build-up of proteins, brain clearance, neuronal activity and inflammation.^7^

## Sleep medications and dementia

Furthermore, when treating sleep disturbances, sleep medications, such as benzodiazepine and non-benzodiazepine hypnotics, melatonin, trazodone, ramelteon, suvorexant, memantine, triazolam, risperidone and galantamine, play a poorly understood role where they may controversially mitigate the risk of Alzheimer's disease.^8^ This management approach, however, is still not well-elucidated, with many medical associations discouraging sleep medications among older adults because of their association with cognitive impairment.^9^ In fact, a study analysing data from a national USA older adult sample found that routine sleep medication use was associated with incident dementia.^10^ Similarly, a meta-analysis has indicated a strong association between excessive polypharmacy and dementia.^11^ Although behavioural therapies are available, sleep management through medication remains disputed, as its influence on dementia is not completely understood.

## Racial and ethnic differences

Sleep disturbances are prevalent, with well-pronounced racial and ethnic differences, among USA middle-age and older adults. Black adults report higher odds of sleep apnoea syndrome, poor sleep quality, short sleep and daytime sleepiness compared with White participants.^12^ Hispanic and Chinese adults report higher odds of sleep disordered breathing and short sleep compared with White adults.^12^ The data, however, is not definitive, as some evidence has shown Black and White individuals reporting a worse score on sleep disturbances relative to Hispanic and Asian individuals.^13^ Asian adult sleep patterns have been less studied, but evidence has shown less sleep disturbances than other racial and ethnic groups.^14^ In addition to sleep disturbances, racial and ethnic differences in dementia risk are also amply documented. Compared with USA White older adults, Black and Hispanic older adults have a disproportionately higher dementia risk.^15,16^ Although the underlying racial and ethnic factors leading to increased dementia risk are not fully understood, epidemiological models outline an interplay between comorbidities, social determinants of health and genetic factors as possible contributors.^17^

## Aims

To our knowledge, no prior research has investigated the relationship between sleep disturbances, race and ethnicity, and dementia risk. In fact, a recent 2023 study examining the association between sleep and dementia risk has called for future research to investigate how the interaction between race/ethnicity and sleep disturbances may influence dementia risk.^1^ Therefore, our present study uses longitudinal prospective data of a national USA older adult sample to (a) explore racial and ethnic disparities in sleep disturbances (sleep-initiation difficulty, sleep-maintenance difficulty, sleep medication usage) in our study cohort, and (b) examine whether race and ethnicity moderates the relationship between sleep disturbances and dementia risk.

## Method

### Data source

We analysed 10 years (2011–2020) of data from the National Health and Aging Trends Study (NHATS), a prospective longitudinal cohort study that collects data from a nationally representative sample of USA Medicare beneficiaries aged ≥65 years. The NHATS oversamples individuals at older ages and Black older adults relative to their representation in the Medicare frame. Our sample consisted of 6284 community-dwelling, dementia-free older adults at baseline in 2011. The authors assert that all procedures contributing to this work comply with the ethical standards of the relevant national and institutional committees on human experimentation and with the Helsinki Declaration of 1975, as revised in 2008. All procedures involving human patients were approved by the SUNY Upstate Institutional Review Board for the Protection of Human Subjects (approval number 1920833-1). NHATS interviewers obtained written informed consent from all respondents.

### Dementia diagnosis

Dementia diagnosis was developed with an NHATS algorithm^18^ that is based on three measures, asked annually: (a) AD8 Dementia Screening Interview,^19^ which assesses memory, temporal orientation, judgement and function; (b) multiple cognitive tests assessing memory (e.g. immediate ten-word recall), orientation (e.g. date) and executive function (e.g. clock drawing test); and (c) self-reported Alzheimer's disease or dementia diagnosis by a doctor. From these measures, we used an NHATS-derived binary variable for a probable dementia diagnosis, which the NHATS has tested to have a reasonably good sensitivity of 65.7% and high specificity of 87.2%.^19^

### Sleep disturbances

During the 10-year period between 2011 and 2020, there were only three questions for sleep disturbances that were asked annually. The first question was sleep-initiation difficulty: ‘In the last month how often did it take you more than 30 min to fall asleep?’. The second question was sleep-maintenance difficulty: ‘In the last month on nights when you woke up before you wanted to get up, how often did you have trouble falling back asleep?’. The third question was sleep medication usage: ‘In the last month how often did you take medication to help you sleep?’.

All sleep disturbance questions were answered as never/not a problem, rarely (once a week or less), some nights (two to four nights a week), most nights (five to six nights a week) or every night (seven nights a week). To create longitudinal sleep disturbance measures, we replicated the approach previously used in a separate study by using these same three sleep measures in the NHATS data.^1^ We first transformed all three sleep disturbances into a binary response: no (never or rarely) or yes (some nights, most nights or every night). For our second step, each binary sleep disturbance variable from every wave was then combined into a longitudinal score, which measured the proportion of years with each respective sleep disturbance before dementia diagnosis or censoring. These longitudinal sleep disturbance scores can have a range from zero to one (or 0 to 100%). For instance, a respondent who entered the study in 2011 and died in 2016 may have reported sleep-initiation difficulty at two waves during the 5-year period they were alive from 2011 to 2015, resulting in a longitudinal sleep-initiation difficulty score that is two-fifths or 40%. This same method was performed for the other two sleep measures as well. These sleep scores precede dementia diagnosis and exhibit a longitudinal measure for each sleep disturbance between 2011 and 2020, or earlier depending on when respondents were diagnosed with dementia or were censored.

### Race and ethnicity

Race and ethnicity was self-reported as either non-Hispanic White (hereafter, White), non-Hispanic Black (hereafter, Black), Hispanic, Asian and other. The ‘other’ category (*n* = 129) combined multiple racial and ethnic groups because of low sample sizes. This group included American Indian (46.5%, *n* = 60), multiracial (4.7%, *n* = 6) and unspecified (48.8%, *n* = 63) respondents.

### Covariates

Regression models were adjusted for self-reported sociodemographic and health characteristics at baseline. Sociodemographic covariates included age, gender (male or female), highest level of education (less than high school, high school or college), total household income, marital status (married or unmarried) and metropolitan residence (metro or non-metro).

Health covariates included self-rated overall health condition (poor, fair, good, very good or excellent), body mass index, activities of daily living (ADL) (no ADL limitations or at least one ADL limitation), proxy respondent, major depressive disorder (using the Patient Health Questionnaire-2), generalised anxiety disorder (using the Generalised Anxiety Disorder-2), heart attack history, hypertension history and diabetes history.

### Analysis plan

Cox proportional hazards models analysed the number of years from dementia-free at baseline (2011) to dementia diagnosis, adjusted for all aforementioned covariates. We censored respondents if they died, dropped out or did not have a dementia diagnosis by the end of the study period. An interaction between race/ethnicity and each of the three sleep disturbances were created, and all variables were entered into one fully adjusted Cox model. Average variance inflation factor was 2.23, indicating no evidence of multicollinearity. Survey sampling weights were applied to ensure that the results obtained were representative of the USA older adult population.

To minimise potential bias from missing data (6.4%) and maximise all completed responses in the data, we utilised multiple imputation by chained equations to create 100 imputed data files for the regression. Sensitivity analyses were conducted to determine the robustness of findings by stratifying the sleep–dementia relationship for each race and ethnicity, comparing results to listwise deletion, and shifting the cut-off of sleep disturbances (some nights versus most nights). All statistical analyses were performed in Stata 18 on MacOS, with two-tailed tests at a 0.05 significance level.

## Results

### Sample characteristics

Among the 6284 respondents, 17.4% (*n* = 1094) developed an incident dementia diagnosis during the 10-year period, with an incidence rate of 29.80 per 1000 person-years (95% CI 28.37–31.30). Incidence rates among each racial and ethnic group were highest for Hispanic older adults (60.35 per 1000 person-years, 95% CI 50.82–71.67), followed by Asian (44.80 per 1000 person-years, 95% CI 30.93–64.88), Black (43.74 per 1000 person-years, 95% CI 39.61–48.29), White (30.0 per 1000 person-years, 95% CI 28.15–31.87) and other (4.96 per 1000 person-years, 95% CI 3.65–6.74) older adults in the USA.

Table 1 describes the characteristics for the sample stratified by race and ethnicity. Average age was 76.5 years, and more than half (57.1%) were female. The majority (62.5%) were college educated, with an average annual income of about $52 000. A slight majority (53.7%) were married, and the majority (81.1%) were living in a metropolitan area.

**Table 1:** Sample characteristics at baseline^a^

	Whole sample (*N* = 6284)	White (*n* = 4394, 69.9%)	Black (*n* = 1311, 20.9%)	Hispanic (*n* = 342, 5.4%)	Asian (*n* = 108, 1.7%)	Other (*n* = 129, 2.1%)
Age, years (range 65–105)	76.5 (7.4)	76.9 (7.5)	75.4 (6.9)	75.9 (7.4)	75.8 (6.3)	76.2 (7.5)
Female, % (*n*)	57.1% (3585)	56.2% (2471)	60.0% (786)	55.6% (190)	54.6% (59)	61.2% (79)
Highest level of education, % (*n*)						
Less than high school	11.6% (727)	8.2% (359)	19.9% (260)	26.0% (89)	9.3% (10)	8.0% (9)
High school degree	25.9% (1623)	28.8% (1265)	21.1% (276)	14.6% (50)	12.0% (13)	16.8% (19)
College degree	62.5% (3916)	63.0% (2769)	59.1% (774)	59.4% (203)	78.7% (85)	75.2% (85)
Household income (US$, thousands)	51.9 (15.3)	59.3 (15.7)	32.4 (62.5)	42.1 (30.8)	39.5 (48.3)	32.3 (24.8)
Married, % (*n*)	53.7% (3373)	58.2% (2557)	38.0% (498)	53.1% (181)	67.6% (73)	50.0% (64)
Metropolitan residence, % (*n*)	81.1% (5093)	77.2% (3393)	90.0% (1180)	90.6% (310)	99.1% (107)	79.8% (103)
Self-rated health (0–4; poor to excellent)	2.2 (1.1)	2.4 (1.1)	1.9 (1.0)	1.7 (1.1)	2.0 (1.1)	2.2 (1.2)
Body mass index, kg/m^2^	27.7 (5.60)	27.3 (5.3)	29.3 (6.4)	28.0 (5.2)	24.8 (5.6)	27.7 (5.7)
ADL limitations, % (*n*)						
None	89.5% (5585)	90.9% (3977)	87.3% (1135)	79.5% (267)	89.6% (95)	86.7% (111)
At least one	10.5% (658)	9.1% (396)	12.7% (165)	20.5% (69)	10.4% (11)	13.3% (17)
No proxy respondent, % (*n*)	98.0% (6161)	98.7% (4335)	98.1% (1286)	95.9% (328)	83.3% (90)	94.6% (122)
Depression, % (*n*)	13.0% (819)	11.2% (488)	17.4% (226)	21.6% (73)	8.4% (9)	18.0% (23)
Anxiety, % (*n*)	11.0% (691)	10.2% (446)	11.8% (154)	19.2% (65)	10.2% (11)	11.9% (15)
History of heart attack, % (*n*)	14.2% (894)	14.7% (646)	12.9% (169)	12.3% (42)	11.2% (12)	19.4% (25)
History of hypertension, % (*n*)	67.1% (4213)	63.6% (2790)	80.2% (1051)	64.6% (221)	68.5% (74)	59.7% (77)
History of diabetes, % (*n*)	24.9% (1565)	20.5% (901)	37.4% (490)	32.5% (111)	25.9% (28)	27.1% (35)

ADL, activities of daily living.

a. Unless otherwise indicated, data are expressed as mean (s.d.).

On average, respondents were in ‘good’ health, but had an average body mass index of 27.7 kg/m^2^, which is considered overweight. The majority had zero ADL limitations (89.5%) and no need for a proxy respondent (98.0%). Among the health conditions, history of hypertension (67.1%) and diabetes (24.9%) were the most frequently reported.

The inclusion of five different racial and ethnic groups in our study inhibits detailed comparisons of sociodemographic and health differences, but are shown in Table 1. In general, noteworthy results include lower annual income for Black (mean $32 400), Hispanic (mean $42 100) and Asian (mean $39 500) respondents compared with White respondents (mean = $59.3 k). Metropolitan residence was higher for Black (90%), Hispanic (90.6%) and Asian (99.1%) respondents compared with White respondents (77.2%). Several chronic diseases, such as diabetes, were also more frequent for Black (37.4%), Hispanic (32.5%) and Asian (25.9%) respondents compared with White respondents (20.5%).

### Bivariate results

Table 2 presents results stratified by race and ethnicity for each sleep disturbance longitudinal score, ranging from 0 to 100% during the 10-year period that respondents were in the study. There were statistically significant differences in average longitudinal score for sleep-initiation difficulty by race and ethnicity (F(4,6187) = 13.75, *P* < 0.001). In particular, sleep-initiation difficulty was more common among Hispanic (53.8%), Asian (53.1%) and Black (50.9%) older adults compared with the whole sample (45.2%).

**Table 2:** Average frequency of sleep disturbances, stratified by race and ethnicity

	Whole sample (*N* = 6284)	White (*n* = 4394, 69.9%)	Black (*n* = 1311, 20.9%)	Hispanic (*n* = 342, 5.4%)	Asian (*n* = 108, 1.7%)	Other (*n* = 129, 2.1%)	ANOVA test
Sleep-initiation difficulties	45.2%	42.7%	50.9%	53.8%	53.1%	42.9%	F(4,6187) = 13.8, *P* < 0.001
Sleep-maintenance difficulties	43.5%	42.8%	45.0%	45.9%	46.4%	42.0%	F(4,6209) = 1.20, *P* = 0.31
Sleep medication usage	20.7%	22.9%	13.7%	20.7%	14.6%	19.6%	F(4,6205) = 17.5, *P* < 0.001

This longitudinal score can have a range from 0 to 100%, which measures the proportion of years with each respective sleep disturbance before dementia diagnosis or being censored. ANOVA, analysis of variance.

Similarly, sleep-maintenance difficulty was more frequent on average among Asian (46.4%), Hispanic (45.9%) and Black (45.0%) older adults compared with the whole sample (43.5%). These group differences, however, were not statistically significant (F(4,6209) = 1.20, *P* = 0.31).

Finally, there were significant differences in average longitudinal score for sleep medication usage by race and ethnicity (F(4,6205) = 17.46, *P* < 0.001). Specifically, sleep medication usage was lower among Black (13.7%) and Asian (14.6%) older adults compared with the whole sample (20.7%)

### Cox regression results

We examined the moderating role of race and ethnicity between sleep disturbances and dementia risk. For sleep-initiation difficulty, there was only a significant interaction for USA Hispanic older adults (adjusted hazard ratio (aHR) 0.34, 95% CI 0.15–0.76, *P* < 0.01) (Table 3). Specifically, compared with White older adults, Hispanic older adults with more frequent difficulty falling asleep within 30 min had a significantly decreased dementia risk. This was supported in a sensitivity analysis after restricting the sample to Hispanic respondents, in which sleep-maintenance difficulty was associated with about a 55% decreased dementia risk (aHR = 0.45, 95% CI 0.24–0.84, *P* = 0.02) (Table 4). There were no significant interactions for all other racial and ethnic groups.

**Table 3:** Race/ethnicity and sleep disturbance association with dementia risk

	Adjusted hazard ratio (95% CI), *P-*value
Racial–ethnic group	
White, non-Hispanic	Reference
Black, non-Hispanic	1.38 (1.06–1.80), 0.02
Hispanic	1.69 (1.17–2.44), <0.01
Asian	1.57 (0.80–3.06), 0.18
Other	0.97 (0.41–2.31), 0.94
Sleep disturbance type	
Sleep-initiation difficulties	1.05 (0.78–1.41), 0.75
Sleep-maintenance difficulties	0.53 (0.39–0.72), <0.01
Sleep medication usage	1.11 (0.88–1.39), 0.39
Sleep-initiation difficulties interaction	
White, non-Hispanic	Reference
Black, non-Hispanic	0.97 (0.59–1.59), 0.90
Hispanic	0.34 (0.15–0.76), 0.01
Asian	0.49 (0.13–1.92), 0.30
Other	2.05 (0.41–10.35), 0.38
Sleep-maintenance difficulties interaction	
White, non-Hispanic	Reference
Black, non-Hispanic	1.20 (0.65–2.23), 0.55
Hispanic	2.68 (1.17–6.13), 0.02
Asian	1.32 (0.57–3.05), 0.51
Other	1.44 (0.23–8.92), 0.69
Sleep medication usage interaction	
White, non-Hispanic	Reference
Black, non-Hispanic	0.84 (0.50–1.41), 0.50
Hispanic	1.02 (0.48–2.15), 0.97
Asian	3.85 (1.64–9.04), <0.01
Other	0.38 (0.12–1.18), 0.09
Weighted population size	32 840 696
Model significance	F(35, 54) = 72.36, *P* < 0.001

All interactions in one model adjusted for age, gender, education, income, marital status, metropolitan residence, overall health condition, body mass index, activities of daily living limitations, proxy respondent, depression, anxiety, heart attack, hypertension and diabetes.

**Table 4:** Association between sleep disturbance and dementia risk, stratified by race and ethnicity

	White, adjusted hazard ratio (95% CI), *P-*value	Black, adjusted hazard ratio (95% CI), *P-*value	Hispanic, adjusted hazard ratio (95% CI), *P-*value	Asian, adjusted hazard ratio (95% CI), *P-*value	Other, adjusted hazard ratio (95% CI), *P-*value
Sleep-initiation difficulty	1.07 (0.81–1.42), 0.62	1.03 (0.66–1.62), 0.89	0.45 (0.24–0.84), 0.02	1.44 (0.15–12.81), 0.71	5.10 (0.33–79.40), 0.23
Sleep-maintenance difficulty	0.53 (0.39–0.72), <0.001	0.66 (0.41–1.07), 0.09	1.29 (0.67–2.48), 0.44	1.14 (0.15–8.40), 0.88	4.56 (0.20–103.81), 0.32
Sleep medication usage	1.07 (0.85–1.34), 0.57	0.88 (0.57–1.35), 0.54	1.19 (0.60–2.34), 0.61	8.16 (1.35–49.46), 0.03	0.20 (0.04–0.90), 0.04
Weighted population size	24 806 831	2 357 841	1 902 249	669 137	642 820
Model significance	F(19, 51) = 41.79, *P* < 0.001	F(19, 47) = 29.58, *P* < 0.001	F(19, 28) = 15.52, *P* < 0.001	F(19, 28) = 15.52, *P* < 0.001	F(19, 9) = 58.80, *P* < 0.001

Model adjusted for age, gender, education, income, marital status, metropolitan residence, overall health condition, body mass index, activities of daily living limitations, proxy respondent, depression, anxiety, heart attack, hypertension and diabetes.

For sleep-maintenance difficulty, there was only a significant interaction for USA Hispanic older adults (aHR = 2.68, 95% CI 1.17–6.13, *P* = 0.02) (Table 3). Compared with White older adults, Hispanic older adults with more frequent trouble falling back to sleep had a significantly increased dementia risk. In a sensitivity analysis of each separate racial and ethnic group, the positive association for sleep-maintenance difficulty was not significant for Hispanic respondents (aHR = 1.29, 95% CI 0.67–2.48, *P* = 0.44); however, there was a significant negative association for White older adults (aHR = 0.53, 95% CI 0.39–0.72, *P* < 0.001) (Table 4). There were no significant interactions for all other racial and ethnic groups.

For sleep medication usage, there was only a significant interaction for USA Asian older adults (aHR = 3.85, 95% CI 1.64–9.04, *P* < 0.01) (Table 3). Compared with White older adults, Asian older adults with more frequent sleep medication usage had a significantly increased dementia risk. This was supported in a sensitivity analysis restricted to Asian respondents, in which using sleep medications was significantly associated with about an 8.2 times increased dementia risk (aHR = 8.16, 95% CI 1.35–49.46, *P* = 0.03). There were no significant interactions for all other racial and ethnic groups.

### Sensitivity analyses

Two additional sensitivity analyses were conducted. First, each sleep disturbance measure in all ten waves in our present analysis has a binary cut-off, with ‘yes’ defined as some nights (two to four nights), most nights (five to six nights) or every night (seven nights). When shifting the cut-off to have more frequent sleep disturbances (most nights or every night), the conclusions remained the same (Supplementary Table 1 available at https://doi.org/10.1192/bjo.2024.814). Second, missing data were imputed in our primary analysis. Results from listwise deletion for incomplete data were the same compared to our current imputed data (Supplementary Table 2).

## Discussion

This study examined the relationship between sleep disturbances, race/ethnicity and dementia risk in a USA older adult sample. For our first research question, we examined whether there were racial–ethnic differences in each of the three sleep disturbances during our 10-year period. Our results indicated older adults who self-identified as Black, Hispanic or Asian had more frequent difficulties with both sleep initiation and maintenance, whereas sleep medication usage was less frequent.

### Racial and ethnic disparities in sleep disturbances

Several hypotheses have been proposed to explain the higher prevalence of sleep disturbances in racial and ethnic minority groups, such as psychosocial stressors, living in disadvantaged neighbourhoods with inopportune noise, higher exposure to ambient light at night and air pollution,^20–25^ all of which are known to disrupt the body's circadian sleep–wake cycles and increase the risk for sleep disturbances.^26^ In addition, discrimination may also contribute to the higher frequency of sleep dysregulation observed among racial and ethnic minority groups.^27^ For instance, after adjusting for social, demographic and mental health covariates, Black and Hispanic individuals who experience perceived discrimination can be 60% more likely to experience sleep difficulties.^28^

In our study, we observed a significant interaction between sleep initiation difficulties and a lower risk of dementia; however, difficulty in sleep maintenance was significantly associated with a higher dementia risk for USA Hispanic older adults. These opposing directional relationships for dementia risk are peculiar, given that Hispanic older adults had a high frequency of both sleep difficulty types throughout the 10-year study period. Furthermore, the interaction between sleep medication usage and dementia risk was only statistically significant for older Asian adults.

### Racial and ethnic disparities in sleep medication usage

Our study observed significant racial and ethnic differences in reporting sleep medication usage. Black, Hispanic and Asian older adults are less likely to report sleep medication usage compared with White older adults. Only a few studies have reported differences in sleep medication usage among different racial and ethnic groups in nationally representative USA populations, and their findings are generally consistent with ours.^29–31^ For example, a cross-sectional analysis of the 2010 Medical Expenditure Panel Survey demonstrated that White adults were twice as likely than Black individuals to use sleep medication. Hispanic participants were 1.6 times more likely than non-Hispanic participants to use sleep medication.^29^ Similarly, according to the National Ambulatory Medical Care Survey 1999–2010, Black and ‘other’ participants were less likely than White participants to use any prescription sleep medication.^30^ A recent prospective cohort study (Health ABC Study) showed that White participants were three times more likely than Black participants to report taking sleep medications often or almost always. Notably, the frequent use of sleep medications was associated with an increased risk of incident dementia among White, but not Black, participants, even after adjusting for sleep duration and disturbances.^31^

Unfortunately, none of these prior studies reported the frequency of sleep medication usage among Asian subpopulations and any potential association with dementia risk. Therefore, the significant dementia risk observed in USA Asian older adults with a more frequent sleep medication usage compared with White older adults contributes to the growing body of literature suggesting that the dementia risk associated with sleep medication may disproportionately affect different racial and ethnic subgroups. However, the mechanism used to observe a relationship between sleep medication usage and dementia for Asian older adults is difficult to elucidate, because of several data constraints: (a) it is unknown if these sleep medications were over the counter, prescription, herbal or traditional; and (b) Asian individuals are inherently a diverse ethnic group, and there may be cultural differences in the perception of using sleep medications between Chinese and Indian households, for example. Therefore, additional research is warranted to understand better the pathways for this phenomenon among the USA Asian older adults.

### Sleep disturbance and race/ethnicity interaction

For our second research question, we examined whether race and ethnicity moderated the relationship between sleep disturbances and dementia risk. Despite the similarly high prevalence of sleep initiation and maintenance among Black, Hispanic and Asian minority subgroups, the significantly higher adjusted risk of incident dementia with difficulty in sleep maintenance in only Hispanic participants is intriguing, and may have several potential contributors. Studies have shown that higher discrimination scores^32^ and perceived stress^33^ are positively associated with disturbed sleep. Notably, after adjusting for confounders, mean discrimination scores were significantly associated with a longer wake duration after sleep onset, but not sleep onset latency.^32^ Also, daily stressors may affect total sleep time but not sleep efficiency, sleep onset latency and wake duration after sleep onset. In contrast, the longer wake duration after sleep onset may predict next-day stressor severity.^34^ These findings suggest that exposure to discrimination and stress may cause difficulties in maintaining, but not initiating sleep, thus contributing to the increased dementia risk observed in the Hispanic subpopulation of our study cohort.

Our hypothesis that exposure to discrimination and stress plays a significant role in the observed higher risk of incident dementia with difficulty in sleep maintenance in Hispanic participants is further supported by three observations. First, nearly 80% of the participants in the Hispanic Community Health Study/Study of Latinos Sociocultural Ancillary Study cohort reported lifetime discrimination exposure.^35^ Second, this study identified chronic moderate/severe stress, ethnic discrimination and acculturation stress as specific sociocultural stressors, with each stressor positively associated with insomnia symptoms, daytime sleepiness and sleep duration.^36^ Finally, these sociocultural stressors may contribute to a higher severity of sleep disturbance among Hispanics than in other subpopulations and, hence, a higher risk of incident dementia. Indeed, data from the five waves of the Health and Retirement Study (2002–2010) showed that Hispanic adults had higher insomnia severity scores compared with non-Hispanic White adults.^37^

Moreover, our results partially resemble the Health and Retirement Study, which found that the trend in higher insomnia severity scores did not decline for USA Hispanic older adults after adjusting the model for accumulated health conditions.^37^ The researchers explained this unexpected trajectory by investigating additional factors such as immigration status, national origin and access to health coverage. However, none of these additional analyses yielded significant findings. As a result, the researchers theorised that acculturation levels, proximity to family members and neighbourhood community engagement may instead play a role.^21^ Ultimately, more research is needed to understand the underlying factors driving the relationship between sleep difficulty and dementia risk among Hispanic older adults. Despite our thorough review of the literature, we were unable to identify factors other than exposure to discrimination and stress that could explain these interaction findings for USA Hispanic older adults. In addition, we could not identify concrete factors in the existing literature that would explain the significantly lower dementia risk among Hispanic older adults with sleep-initiation difficulty compared with White older adults. One potential contributor could be reported differences in the prevalence of very short (<5 h), short (5–6 h) and long (≥9 h) sleep duration based on subgroups defined by race and ethnicity, which have all been associated with a higher risk of incident dementia.^38–40^ In the National Health and Nutrition Examination Survey 2007–2008 cohort, Whinnery et al reported that Hispanic subgroups had a lower relative risk of very long sleep compared with White and Black participants, albeit with a higher relative risk of very short sleep, compared with White participants.^41^ Although Black participants had a higher relative risk of short sleep duration compared with White participants, no significant risk was noted for Hispanic subpopulations.^41^ Similarly, in the 2004–2014 National Health Interview Survey waves, a significantly lower percentage of Hispanic participants reported long sleep duration than White and Black participants, with no differences in very short and short sleep durations. However, a significantly greater proportion of Black participants reported very short and short sleep durations compared with White participants.^42^ Similar trends have been reported in the Alameda County Health and Ways of Living Study^43^ and the Chicago Area Sleep Study.^44^ Thus, it is plausible that a lower prevalence of long sleep duration offsets some of the dementia risk posed by difficulty initiating sleep in the Hispanic population in our study cohort.

### Strengths and limitations

There are several limitations to our study. First, there is no psychometric testing data available for our self-reported sleep disturbance measures. Based on our sensitivity analysis, however, shifting the frequency of sleep disturbances did not alter any findings. Second, there may be heterogeneity in the sleep–dementia relationships within the four broad racial and ethnic groups we examined. Examining these subgroup distinctions was impeded by small samples sizes, but would be worthwhile in future research. Third, establishing temporality between sleep disturbance and dementia onset is limited because of the long latency of cognitive impairment. For example, respondents reporting sleep disturbances may already have dementia-related neuropathology present. Fourth, the NHATS data does not include specific sleep parameters such as total sleep duration, sleep onset latency, sleep efficiency, sleep quality and wake after sleep onset; sleep staging parameters such as slow-wave sleep, non-REM and REM; or the dose, duration and type of sleep medication used to allow for a comprehensive analysis. Finally, although there is a large number of sociodemographic and health variables in our adjusted models, there is potential residual confounding from other variables associated with dementia risk, such as cardiovascular disease and stroke history.

Despite these limitations, to our knowledge, this study is an important contribution to the field as it presents the first analyses examining the intersection between race and ethnicity, sleep disturbances and dementia risk, using longitudinal prospective data with a nationally representative USA older adult sample. Future research is needed with larger samples to determine whether there are certain sociodemographic factors (e.g. age and education) that may be contributing to these differential effects of sleep and dementia by race and ethnicity.

In summary, this study highlights racial and ethnic disparities in sleep disturbances by using a nationally representative USA older adult sample. On average, Black, Hispanic and Asian older adults experience more frequent sleep disturbances and less frequent sleep medication usage than White individuals. The relationship between sleep disturbances and dementia are also moderated by race and ethnicity. Sleep medication usage among Asian older adults is associated with increased dementia risk, whereas sleep-initiation difficulty among Hispanic older adults is associated with decreased dementia risk. Overall, the study provides valuable insights into the complex relationships between race/ethnicity, sleep disturbances and dementia risk, warranting further research to investigate these mechanisms.

## Supporting information

Wong and Grullon supplementary material Wong and Grullon supplementary material

## Data Availability

The sensitive data used in this study may be obtained through an application from the National Health and Aging Trends Study (https://nhats.org/).
