# Supplementary material for: Sleep disturbances and racial–ethnic disparities in 10-year dementia risk among a national sample of older adults in the USA
Source: BJPsych Open. 2024 Dec 4;10(6):e219. doi: 10.1192/bjo.2024.814 (PMC11698159; doi:10.1192/bjo.2024.814)
Supplement: Wong and Grullon supplementary material [file S2056472424008147sup001.docx]

Supplemental Table 1. Race-Ethnicity and Sleep Disturbance Association with Dementia Risk with Sleep Disturbances at Least Most Nights

|  | Adjusted Hazard Ratio (95% CI), *P* |
| --- | --- |
| Racial-Ethnic Group |  |
| White, non-Hispanic | reference |
| Black, non-Hispanic | 1.54 (1.27-1.87), <.001 |
| Hispanic | 1.77 (1.29-2.43), <.01 |
| Asian | 1.25 (0.63-2.49), .52 |
| Other | 1.54 (0.45-5.25), .48 |
| Sleep Disturbance Type |  |
| Sleep-initiation difficulties | 1.34 (1.01-1.78), .04 |
| Sleep-maintenance difficulties | 0.89 (0.61-1.29), .52 |
| Sleep medication usage | 0.98 (0.75-1.28), .88 |
| Sleep-initiation Difficulties Interaction |  |
| White, non-Hispanic | reference |
| Black, non-Hispanic | 0.56 (0.34-0.93), .03 |
| Hispanic | 0.27 (0.10-0.77), .01 |
| Asian | 0.57 (0.12-2.79), .48 |
| Other | 3.47 (0.12-100.25), .46 |
| Sleep-maintenance Difficulties Interaction |  |
| White, non-Hispanic | reference |
| Black, non-Hispanic | 1.78 (0.91-3.48), .09 |
| Hispanic | 2.86 (1.26-6.50), .01 |
| Asian | 1.94 (0.28-13.31), .49 |
| Other | 0.15 (0.00-75.06), .54 |
| Sleep Medication Usage Interaction |  |
| White, non-Hispanic | reference |
| Black, non-Hispanic | 0.93 (0.52-1.66), .80 |
| Hispanic | 0.95 (0.36-2.52), .92 |
| Asian | 4.34 (1.57-11.97), <.01 |
| Other | 0.18 (0.01-3.32), .25 |
|  |  |
| Weighted Population Size | 28,562,633 |
| Model Significance | F(36, 21) = 25.38, *P* < .001 |

Note. All interactions in one model adjusted for age, sex, education, income, marital status, metropolitan residence, overall health condition, body mass index, activities of daily living limitations, proxy respondent, depression, anxiety, heart attack, hypertension, and diabetes.

# Supplemental Table 2. Race-Ethnicity and Sleep Disturbance Association with Dementia Risk via Listwise Deletion

|  | Adjusted Hazard Ratio (95% CI), *P* |
| --- | --- |
| Racial-Ethnic Group |  |
| White, non-Hispanic | reference |
| Black, non-Hispanic | 1.40 (1.07-1.83), .02 |
| Hispanic | 1.72 (1.15-2.57), <.01 |
| Asian | 1.25 (0.44-3.58), .67 |
| Other | 1.45 (0.50-4.23), .49 |
| Sleep Disturbance Type |  |
| Sleep-initiation difficulties | 1.05 (0.78-1.41), .75 |
| Sleep-maintenance difficulties | 0.53 (0.39-0.72), <.001 |
| Sleep medication usage | 1.11 (0.88-1.39), 0.39 |
| Sleep-initiation Difficulties Interaction |  |
| White, non-Hispanic | reference |
| Black, non-Hispanic | 0.96 (0.56-1.64), .87 |
| Hispanic | 0.28 (0.12-0.63), <.01 |
| Asian | 0.54 (0.11-2.58), .43 |
| Other | 7.27 (0.40-131.49), .18 |
| Sleep-maintenance Difficulties Interaction |  |
| White, non-Hispanic | reference |
| Black, non-Hispanic | 1.25 (0.67-2.33), .48 |
| Hispanic | 3.28 (1.41-7.65), <.01 |
| Asian | 2.12 (0.77-5.84), .14 |
| Other | 0.13 (0.01-1.85), .13 |
| Sleep Medication Usage Interaction |  |
| White, non-Hispanic | reference |
| Black, non-Hispanic | 0.84 (0.50-1.41), .50 |
| Hispanic | 0.99 (0.42-2.32), .99 |
| Asian | 2.37 (1.07-5.28), .04 |
| Other | 0.32 (0.03-3.69), .35 |
|  |  |
| Weighted Population Size | 28,562,633 |
| Model Significance | F(36, 21) = 25.43, *P* < .001 |

Note. All interactions in one model adjusted for age, sex, education, income, marital status, metropolitan residence, overall health condition, body mass index, activities of daily living limitations, proxy respondent, depression, anxiety, heart attack, hypertension, and diabetes.
